# Translating Behavior Change Principles Into a Blended Exercise Intervention for Older Adults: Design Study

**DOI:** 10.2196/resprot.9244

**Published:** 2018-05-02

**Authors:** Sumit Mehra, Bart Visser, Tessa Dadema, Jantine van den Helder, Raoul HH Engelbert, Peter JM Weijs, Ben JA Kröse

**Affiliations:** ^1^ Applied Psychology Faculty of Applied Social Sciences and Law Amsterdam University of Applied Sciences Amsterdam Netherlands; ^2^ Digital Life - CREATE-IT Applied Research Faculty of Digital Media and Creative Industries Amsterdam University of Applied Sciences Amsterdam Netherlands; ^3^ Informatics Institute Faculty of Science University of Amsterdam Amsterdam Netherlands; ^4^ Amsterdam Center for Innovative Health Practice Faculty of Health Amsterdam University of Applied Sciences Amsterdam Netherlands; ^5^ Faculty of Sports and Nutrition Amsterdam University of Applied Sciences Amsterdam Netherlands; ^6^ Department of Rehabilitation Amsterdam Movement Sciences Academic Medical Center Amsterdam Netherlands; ^7^ Department of Nutrition and Dietetics Internal Medicine VU University Medical Center Amsterdam Netherlands

**Keywords:** frail elderly, aged, activities of daily living, exercise, telemedicine, remote consultation, mobile devices, tablet computers, behavior control, health behavior, treatment adherence and compliance

## Abstract

**Background:**

Physical activity can prevent or delay age-related impairments and prolong the ability of older adults to live independently. Community-based programs typically offer classes where older adults can exercise only once a week under the guidance of an instructor. The health benefits of such programs vary. Exercise frequency and the duration of the program play a key role in realizing effectiveness. An auxiliary home-based exercise program can provide older adults the opportunity to exercise more regularly over a prolonged period of time in the convenience of their own homes. Furthermore, mobile electronic devices can be used to motivate and remotely guide older adults to exercise in a safe manner. Such a blended intervention, where technology is combined with personal guidance, needs to incorporate behavior change principles to ensure effectiveness.

**Objective:**

The aim of this study was to identify theory-based components of a blended intervention that supports older adults to exercise at home.

**Methods:**

The Medical Research Council framework was used to develop the blended intervention. Insights from focus group, expert panels, and literature were combined into leading design considerations.

**Results:**

A client-server system had been developed that combined a tablet app with a database in the cloud and a Web-based dashboard that can be used by a personal coach to remotely monitor and guide older adults. The app contains several components that facilitate behavior change—an interactive module for goal setting, the ability to draw up a personal training schedule from a library containing over 50 exercise videos, progress monitoring, and possibilities to receive remote feedback and guidance of a personal coach.

**Conclusions:**

An evidence-based blended intervention was designed to promote physical activity among older adults. The underlying design choices were underpinned by behavior change techniques that are rooted in self-regulation. Key components of the tablet-supported intervention were a tailored program that accommodates individual needs, demonstrations of functional exercises, monitoring, and remote feedback. The blended approach combines the convenience of a home-based exercise program for older adults with the strengths of mobile health and personal guidance.

## Introduction

### Background

Physical activity (PA) is vital to a healthy life. A sedentary lifestyle is associated with numerous health-related problems such as obesity, diabetes, cardiovascular diseases, various forms of cancer, and depression [1,2]. Furthermore, for older adults, PA can prevent or delay the onset of functional impairments and prolong the ability to live independently [3]. Due to these well-acknowledged health benefits, community-based PA programs have spawned across the world [4,5].

A prototypical example of such a program that has been running for over 35 years in The Netherlands is “More Exercise for Seniors,” abbreviated as MBvO in Dutch. Weekly, 300,000 older adults exercise in a group under the guidance of an instructor. A study evaluating the effects of this specific program, however, concluded that exercising once a week is not sufficient [6]. Studies show a higher frequency and a longer exercise duration is needed to capitalize on the health benefits of PA. At least 5 days a week 30 min of moderate-intensity PA is recommended [7,8], and additional weekly strength and balance exercises prevent the decline of muscle mass and flexibility of older adults [9,10].

This raises the question of how older adults can be stimulated to achieve the recommended levels of PA. First, convenience plays a role. Exercising in a community center several times a week is difficult to achieve due to the cost, time, and effort needed to travel [11]. To attain the recommended intensity, a home-based exercise program could prove a useful addition to a community-based program—in the convenience of their home, older adults can continue the exercises they have learnt during the weekly community classes.

Second, older adults need support in following an exercise program. In community classes, an instructor chooses which exercises are suited for the participants, provides instructions how they can be performed safely, and motivates the older adults to persevere. However, technology is increasingly being used for these functions. The potential to reach a large population and low costs are reasons for its emerging popularity. Various reviews indicate that technology-supported interventions can increase the effectiveness of exercise programs [12-17]. Most of the reported studies used websites to deliver the intervention. More recently, mobile health (mHealth), the use of mobile devices to deliver health solutions, however, has become popular [18]. In particular, for older adults, the use of tablets seems promising. Studies show that due to its large touchscreen, older adults are able to operate tablets better than personal computers [19,20] or smartphones [21]. The usability of tablets may be the reason for its increasing popularity in the United States [22] and The Netherlands [23,24] and its use in mHealth interventions for older adults [25].

Nevertheless, there are also limitations in the scope of mHealth. Automated feedback and guidance (ie, avatar coach) do not correspond well with the subtlety and social support that a person can provide [26]. A blended intervention, where personal guidance by a coach is matched with the possibilities technology can deliver, can be an effective approach. The blended intervention might be a useful extension of traditional community-based PA programs. It can increase the exercise frequency of such programs while relying on an existing infrastructure, such as the availability of instructors and the social support of peers.

### Objective

As part of the VITAMIN (VITal Amsterdam elderly IN the city) and MOTO-B (MOtivating Technology for Older adults’ Behavior) projects that aim to increase the vitality of older adults, we conceived a tablet-supported intervention to increase PA in older adults that currently participate in a community-based program by combining the convenience of a home-based program, the potential of mHealth, and the strengths of personal guidance. To develop an effective intervention, the following research question was addressed: How can theoretical principles and scientific evidence on behavior change be translated into features of a tablet-supported intervention to increase the PA levels of older adults?

The effectiveness of the intervention will be determined with a randomized control trial (RCT) that is currently ongoing. The procedure of the RCT is detailed in a protocol study [27]. In this study, we describe the design process that led to the blended intervention. The study meets the plea to transparently report how behavioral change interventions are developed [28]. In typical effect studies, either no theoretical underpinnings are provided or are loosely mentioned without giving a detailed report about how they have led to specific design and implementation choices. By sharing the process that led to the design, we attempt to contribute to this field.

## Methods

### The Medical Research Council Framework

To develop the blended exercise program, insights were drawn from scientific literature as well as from practice-based expertise. On the basis of the Medical Research Council (MRC) framework [29], the following steps were undertaken:

- Step 1: Identify attitudes of older adults
- Step 2: Identify scientific evidence and formulate design considerations
- Step 3: Identify requirements of the blended intervention
- Step 4: Design functional components
- Step 5: Implement components of the blended intervention.

After developing the intervention, the MRC framework recommends testing the intervention procedures and assessing the effectiveness in a controlled manner. These validation measures are discussed in the last section of the paper in light of follow-up studies.

### Step 1: Identify Attitudes of Older Adults

Before developing the blended intervention, 8 focus groups were held with 48 older adults currently participating in the weekly MBvO community-based exercise classes. The aim was to explore the need for a blended intervention by investigating the attitudes of older adults toward an additional home-based exercise program and the possibility of supporting technology. The results show that participants recognized the benefits of doing home-based exercises. They had, however, also concerns regarding guidance, safety, and adherence to a home-based exercise program. The majority were open toward technology that could support them on those aspects, though some of them lacked the confidence to operate technical devices (see [30] for more details about the focus group study). Those insights fed into further development of the blended intervention.

### Step 2: Identify Scientific Evidence and Formulate Design Considerations

To identify relevant literature, the ACM, IEEE, Google Scholar, PsycINFO, and PubMed databases were consulted. A combination of the following search terms was used—(“older adults” OR seniors OR elder*), (“physical activity” OR exercise), (app OR internet OR web OR “mobile phone” OR smartphone OR tablet OR mHealth OR eHealth OR technology), and (“behavior change” OR adoption OR prevalence OR use OR attitude OR acceptance OR intent*). Forward and backward references were screened, and the recommender feature of Mendeley Reference Manager was used to identify additional sources. Studies that were assessed to be relevant, where precedence was given to reviews and meta-analysis, were selectively discussed with supervisors and peers from varying disciplines.

On the basis of the scientific evidence, 3 design considerations were formulated that address the issues identified in step 1.

#### Design Consideration 1: Functional Exercises

As people age, they lose muscle strength, flexibility, balance, and endurance. The decline across these 4 domains decreases their ability to perform activities of daily living (ADL); for instance, getting up from a chair, carrying groceries, or opening a jar [8]. A functional training program has shown to be more effective to counter this decline than general PA (ie, walking) or resistance training (ie, exercises with dumbbells) [31,32]. Functional training consists of exercises across the varying domains and is specifically designed to improve the performance of ADL. A functional exercise not only targets a daily task but also mimics its pattern. For instance, a functional exercise aiming to improve the mobility of older adults may contain exercises of walking up and down the stairs several times. Due to this close proximity to everyday life, older adults can integrate it autonomously into their daily routine. Furthermore, due to the resemblance, older adults recognize the relevance of the exercises more easily, which in turn improves the adherence to the training [33].

#### Design Consideration 2: Behavior Change

**Figure 1:**
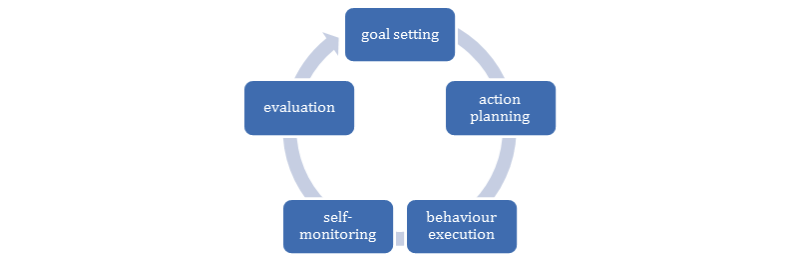
Behavior change through self-regulation.

To regularly perform exercises at home, older adults have to develop new habits. The needed behavior change is hard to achieve, as indicated by the large part of the population that does not meet the recommended levels of PA and the low adherence rate to PA programs that seek to increase this [34,35]. Insights from the behavioral sciences can improve the effectiveness of these interventions. Notably, Michie et al [36] developed a taxonomy of 91 behavior change techniques (BCTs) that were synthesized from a wide body of evidence and afterwards refined to the CALO-RE taxonomy [37]. Techniques that are associated with the self-regulation of behavior appear particularly effective: goal-setting and self-monitoring [38]. In addition, techniques that increase self-efficacy, such as action planning and demonstrating behavior, are also shown to be effective [39]. When these techniques are combined with an evaluation phase in an iterative manner, it corresponds with the widely adopted control theory [40]. See Figure 1 for a schematic representation of the behavior change process adopted in this paper.

#### Design Consideration 3: Blended Technology

Although the effectiveness of eHealth and mHealth to increase PA has been debated [41], 2 common success factors emerge from the literature.

First of all, feedback and guidance play a key role in enriching the various phases of the control theory. Several reviews underline the effectiveness of combining home-based exercise programs for older adults with (remote) guidance [39,41-44]. Geraedts et al [44] identified numerous studies that show higher adherence for home-based exercise intervention programs that included remote feedback. For instance, King et al [45] found the adherence rate of the home-based exercise program initially to be higher than the center-based program, but in a follow-up study [46], they reported a strong drop in adherence levels when the phone calls were ceased according to protocol after 1 year. Geraedts et al plead that PA interventions for older adults should utilize technology to support remote feedback and personal guidance.

The second success factor identified was tailoring. Krebs et al [14] concluded from a meta-analysis of various health behavior change studies, of which 25 targeted PA, that interventions with computer-tailored exercise programs were more effective than the control groups, with effect sizes varying from small to moderate. On average, 43% of participants in the eHealth groups adhered to the PA recommendations compared with 34% in the control groups. Furthermore, iterative tailoring was associated with a larger effect size than tailoring that was only done on the basis of baseline measurements, and this effect was stronger for longitudinal outcomes.

The importance of these 2 factors is also reflected by practice-based findings. The previously described focus group study (step 1) indicated that prospective participants believed additional home exercises would be a useful addition to the group-based classes, but also had worries about the motivation and adherence to such a program [30]. They value the personal guidance and feedback that the instructor provides during the weekly group-based classes. The majority of the participants were open to the idea of using supporting technology in doing exercises at home, though there were concerns if they were able to operate it.

### Step 3: Identify Requirements of the Blended Intervention

By consulting experts from health sciences (requirement 1 and 3) and behavior science (requirement 2 and 4), leading requirements were formulated.

#### Requirement 1: Comprehensiveness

Following design consideration 1, the app should contain functional exercises that vary across domains and difficulty level that can be performed safely in a home setting. The exercises should target the strength, endurance, flexibility, and balance of older adults.

#### Requirement 2: Effectiveness

Following design consideration 2, the app should facilitate behavior change by supporting self-regulation.

#### Requirement 3: Adaptability

Following design consideration 3, the user should be able to create and customize a personal training schedule according to individual needs. Users should be able to increase or decrease the complexity as well as the physical load of the exercise.

#### Requirement 4: Remote Guidance

Following design consideration 3, the app should facilitate remote guidance by a personal coach to motivate and counsel users.

The identified requirements were discussed with practitioners who were involved with the community-based exercise programs for older adults. They confirmed the relevance of the requirements.

## Results

After identifying the needs, design considerations, and requirements (step 1 to 3), consultation of behavioral scientist, computer scientist, and designers resulted in the design and implementation of the blended intervention (steps 4 and 5).

### Step 4: Design Functional Components

By consulting a physical therapist and behavioral scientist, the 4 requirements that were formulated were translated into the components described below.

#### Comprehensiveness: Exercise Library

Users can browse through a library of 17 functional exercises. For each exercise, 3 variations are available that differ in complexity, amounting to a total of 51 exercise variations. Each exercise variation contains a video demonstration with a voice-over for verbal instructions, a factsheet with written instructions, and background information. The instructions stress how the exercises can be performed safely.

#### Effectiveness and Adaptability: Goal Setting

When using the app for the first time, older adults start out by filling in an interactive series of questions. First, they select the activities they value from a set of predefined ADLs. They then prioritize the selected ADLs by ranking these into a top 5 list. Finally, in the last step, the app recommends a number of exercises that match their goals. The user has the possibility to either add those exercises to their personalized exercise program or to ignore the suggestions. Moreover, users can commit to personal goals that they formulate themselves (free-choice alternatives).

#### Effectiveness and Adaptability: Action Planning

The exercise can subsequently be added to the personal training schedule of the user. When adding the exercises, users select the variation and the day they would like to perform the exercise. Optionally, they can set a reminder for a specific time to be alerted.

#### Effectiveness and Adaptability: Behavior Execution

Before performing the exercises as scheduled by the action planning app, users have the opportunity to watch a video in which an older adult demonstrates the exercise along with a voice-over explaining various aspects. Furthermore, before execution, they can alter the physical load with 3 parameters—the duration of the exercise (30, 60, or 90 seconds), the number of repetitions (1, 2, or 3), and the intensity level (1, 2, or 3). During execution of the exercise, users are supported by a countdown timer that keeps track of the duration and repetitions.

#### Effectiveness and Adaptability: Self-Monitoring

After an exercise is performed, users are asked to rate the exercise with a visual analogue scale (slider) on 3 aspects: (1) the complexity of performing the exercise, (2) the effort it took, and (3) the likeability of the exercise. After rating the exercise, it is marked as completed. With checkmarks and progress bars, users can view their progress at a glance.

#### Remote Guidance: Videoconference

Users can make a video call to a personal coach. This coach has remote access to the personal schedule, the exercise parameters, and the ratings of the user. In dialogue with their personal coach, users can reflect on their progress by comparing their goals with their performance. If needed, they can adjust either their goals or the training schedule.

By employing creative brainstorming techniques (eg, scenarios, personas, wire-frames) during sessions with physical therapists, behavior scientists, and interaction designers, BCT defined by CALO-RE (Coventry, Aberdeen & London–Refined taxonomy) [37] were translated into envisioned functions of the tablet app. See Table 1 for the mapping.

### Step 5: Implement Components of the Blended Intervention

#### Functional Exercises

The exercises were developed by a team of human movement scientists and physiotherapists. During the development of the program, active involvement of the older adults, PA trainers, and health professionals was arranged to guarantee that all exercises were understandable, feasible, and could be performed safely. Exercises were first piloted under supervised conditions in the group exercise setting, then under supervision at the older adults’ homes, and finally by the older adults without direct supervision. During this process, the exercises and instructions were fine-tuned to achieve optimal functioning.

#### Envisioned Use Case During Randomized Controlled Trial

**Figure 2:**
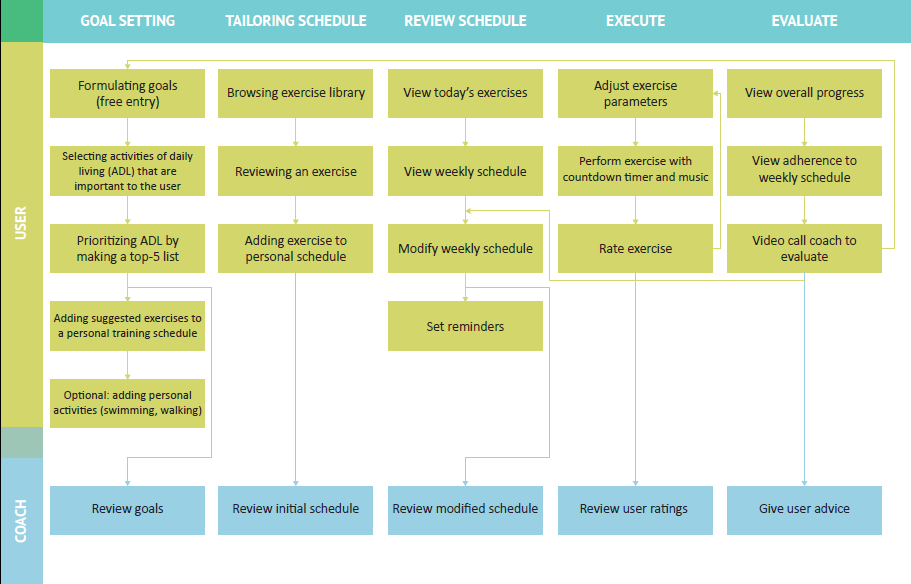
Overview of the use of the tablet app.

In line with the MRC framework, the blended intervention will be tested with an RCT. During this study, older adults are screened for eligibility, and a research coordinator assigns a personal coach to a user. The coach hands out the tablet to the user, along with a short written instruction on how to operate the device. Moreover, a short demonstration is given, and the user can try out the app himself. Then, the user starts by setting goals and drawing up a personal training schedule, assisted by the coach. After this, the user can perform the exercises independently at home. All activity on the tablet is sent to the server (goals, training schedule, and exercise ratings), which can be remotely monitored by the coach. At agreed times, the user seeks guidance of the personal coach by starting a video call within the app. User and coach reflect on the progress, and if needed, the user modifies his goals or training schedule afterward. This process can be done iteratively to support the self-regulation cycle. See Figure 2 for an overview of the use case.

#### Software Architecture

**Figure 3:**
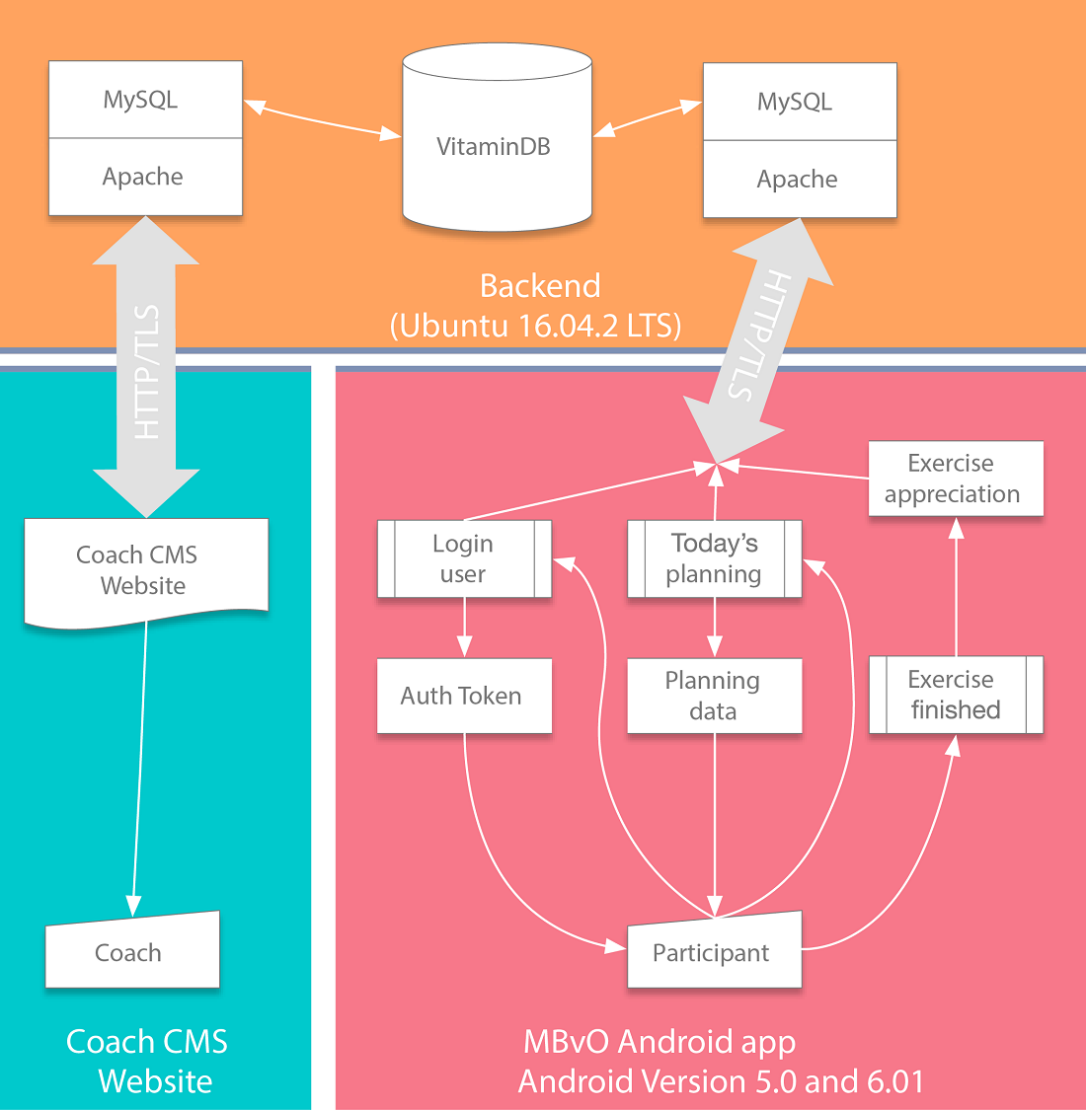
Information Technology architecture. MySQL: open-source relational database management system; APACHE: open-source web server; VITAMIN DB: database containing all relevant data; CMS: content management software; MBvO: More Exercise for Seniors; Android: operating system for tablet computers; Ubuntu: operating system for the server; HTTP/TLS: encrypted network traffic.

The functional components described in the previous step were implemented in a client-server system consisting of an app that was optimized for a 10-inch Android tablet, a back-end for data storage and communication, and a Web-based dashboard to establish communications with the human coaches. See Figure 3 for an overview.

On the tablet, users can set goals, view video demonstrations, create and modify personal training schedules, and rate exercises. The goals, training schedule, and exercises ratings of the user are securely sent to the back-end server and stored in a database. Personal coaches assigned to the users can login on a secured website and view the goals, training schedule, and exercise ratings of the user.

#### User Interface

**Figure 4:**
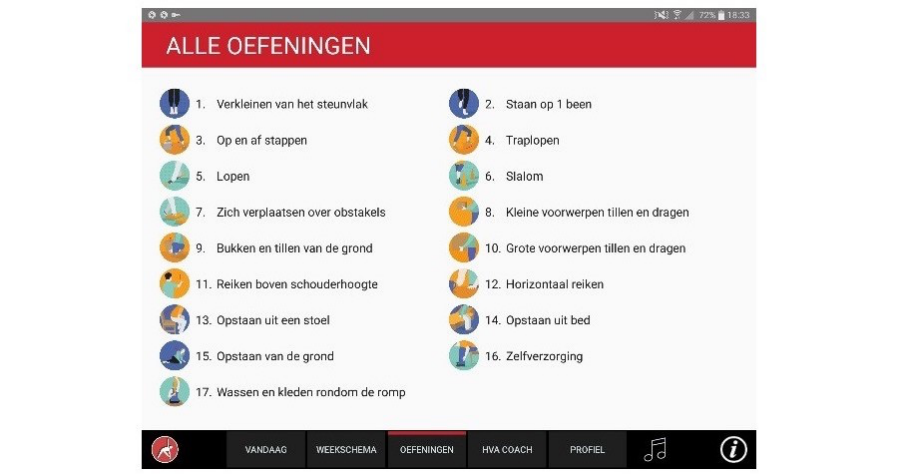
Exercise library.

**Figure 5:**
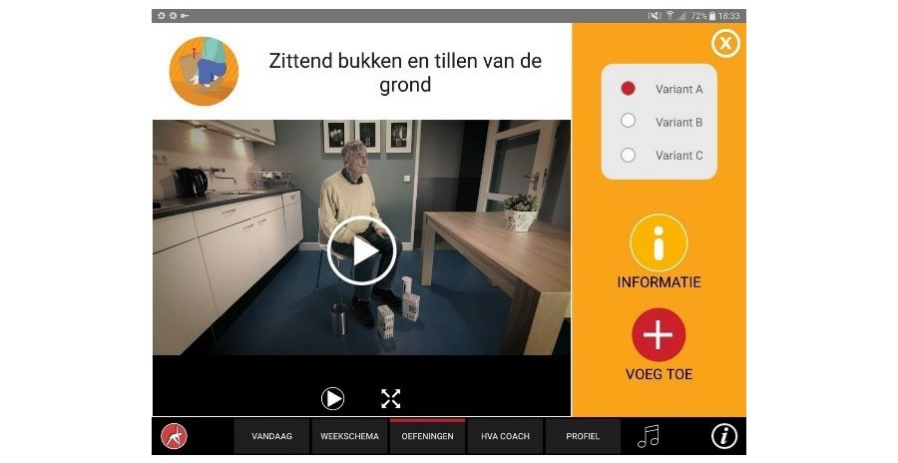
Selecting exercise variation.

**Figure 6:**
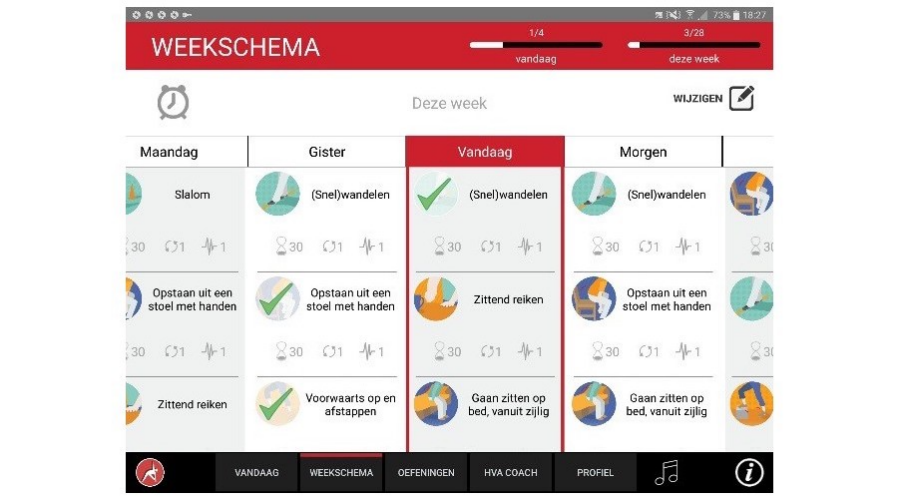
Personal training schedule.

**Figure 7:**
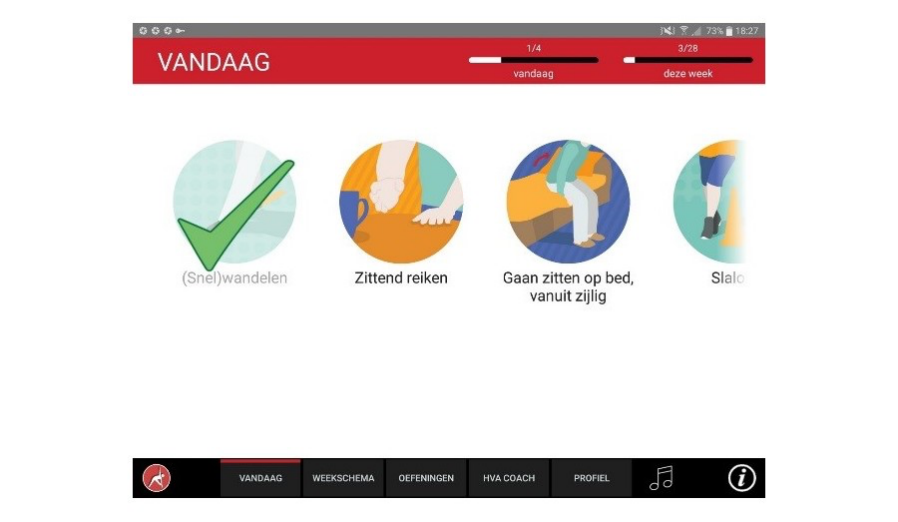
Today’s program.

**Figure 8:**
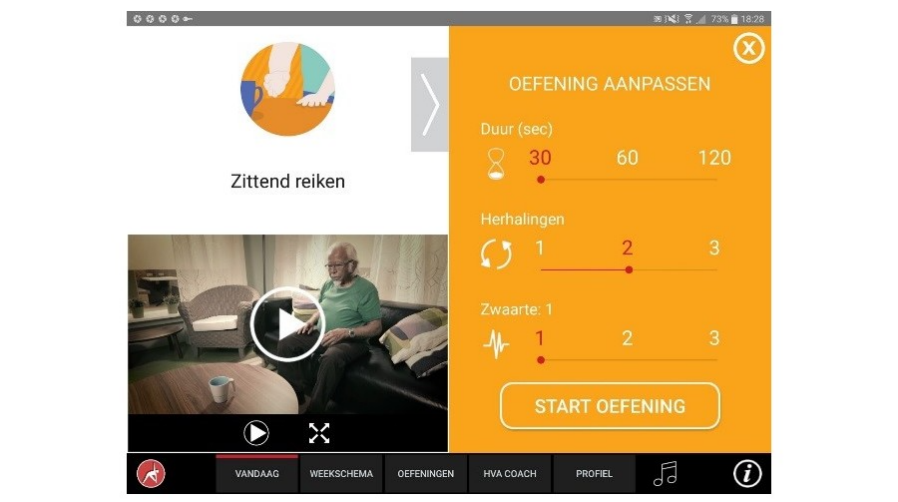
Modifying execution parameters.

**Figure 9:**
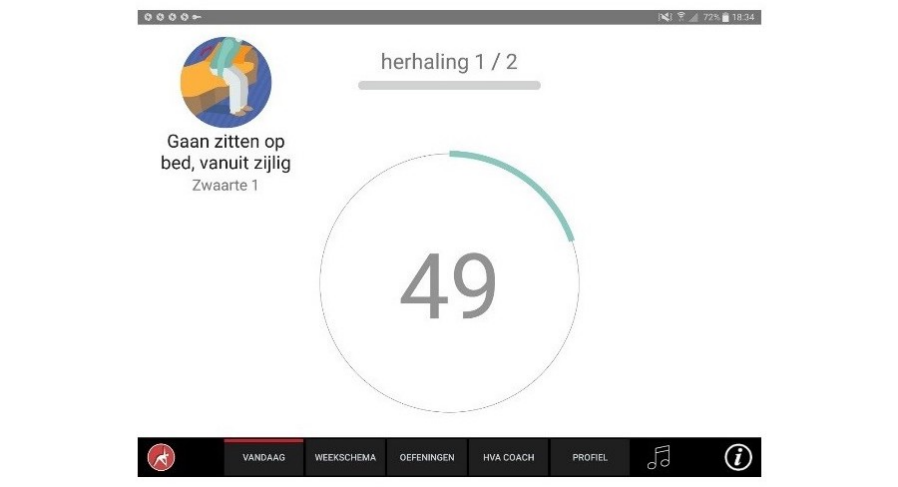
Countdown timer during executing.

**Figure 10:**
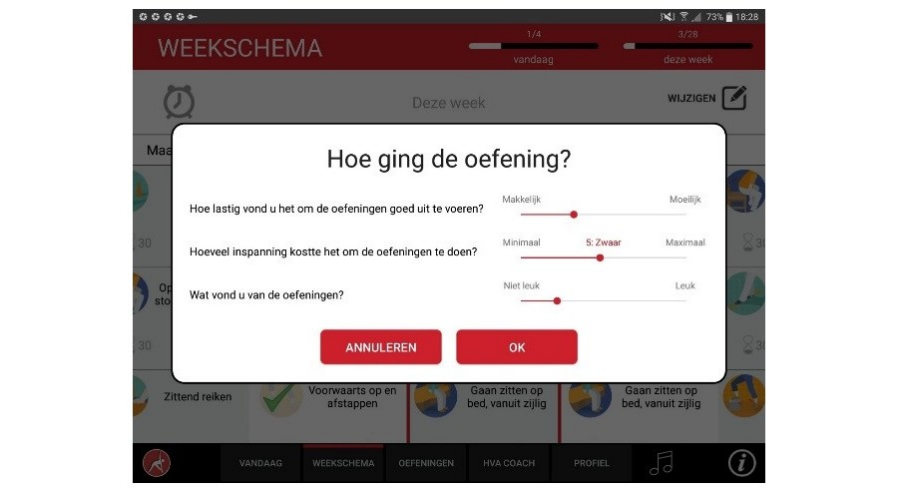
Rating an exercise.

**Figure 11:**
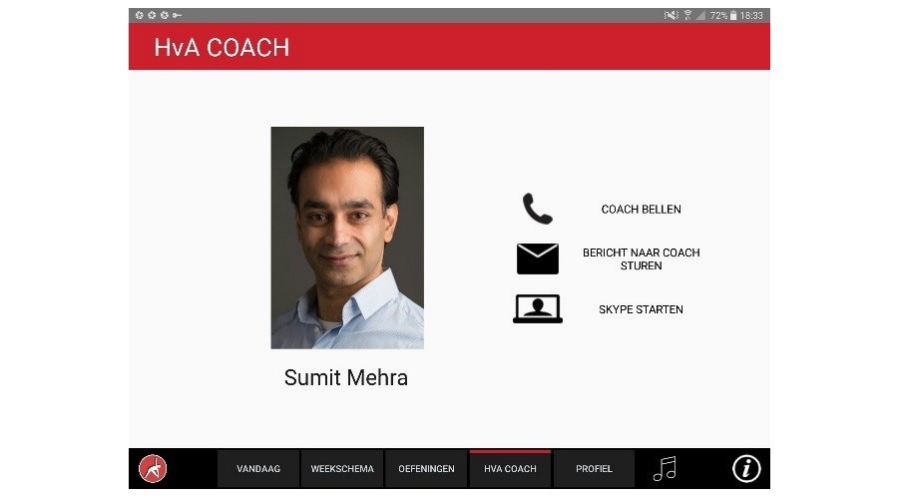
Initiating video call to coach.

To ensure the usability for older adults with no prior experience with mobile devices, simplicity was the guiding principle. Information was layered in various tabs, a metaphor based on an agenda or a Rolodex that older adults are familiar with was used. Furthermore, the visual design was also kept simple. The interface was kept clean with a limited number of elements. Exercises were represented by pictograms that could be viewed at a glance. Large font sizes and contrasting colors were used to ensure readability. To validate the interface, a small-scale usability test was done with 3 prospective users. Various minor modifications were made to improve the usability. See Figures 4-11 for an impression of the resulting user interface.

**Table 1:** Mapping between behavior change techniques and functions of the tablet app.

BCTs^a^ as defined by CALO-RE^b^	Function of the tablet app
Identifying barriers or problem resolution	Videoconference with personal coach (intake)
Goal setting	Prioritize activities of daily living and formulating SMART (Specific, Measurable, Attainable, Realistic and Timely) goals
Setting graded tasks	Three variations of each exercise; before 3 execution parameters can be altered
Action planning	Tailored daily and weekly schedules
Prompt practice	Reminders or alarm
Instruction on how to perform the behavior	Voice-over instructions during video, written instructions in the specification sheet of each exercise, and countdown timer during exercises
Demonstrate behavior	Video depicting an older adult demonstrating how the exercise should be performed
Self-monitoring	Marking exercises as done; rating exercises on effort, complexity, and likeability
Provide feedback on performance	Videoconference with personal coach
Review of behavioral goals	Video conference with personal coach; modification of weekly schedule
Review of outcome goals	Video conference with personal coach; modification of SMART goals
Informing when and where to perform the behavior	Videoconference with personal coach
Environmental restructuring	Videoconference with personal coach
Training to use prompts	Videoconference with personal coach
Motivational interviewing	Videoconference with personal coach
Generalization of target behavior	Videoconference with personal coach
Facilitate social comparison	Weekly face-to-face classes
Plan social support	Weekly face-to-face classes

^a^BCT: behavior change techniques.

^b^CALO-RE: Coventry, Aberdeen & London–Refined taxonomy.

## Discussion

### Principal Findings

By following the MRC framework, a novel intervention has been developed to perform functional exercises at home. It is designed for older adults currently participating in weekly community classes to increase the frequency, duration, and intensity of exercises in a safe and convenient manner in a familiar setting. With a tablet, a customized training schedule can be compiled that matches the personal goals of the user. Furthermore, for motivation and advice, the tablet facilitates remote guidance by a personal coach. Moreover, older adults not participating in community exercise programs can use the tablet autonomously, albeit without the auxiliary guidance of a personal coach. All the components of the blended intervention have been carefully selected and are based on the behavior change theory.

### Contribution and Related Work

There is a wide body of evidence that health interventions that support self-regulation in the general adult population are effective. For older adults, however, there are mixed results. French et al [47] found, in a systematic review, that interventions containing goal-setting and self-monitoring were remarkably associated with lower levels of PA in older adults. They suggest this may be caused by a decline in executive functioning associated with aging. Self-regulation requires cognitive effort. In the systematic review of Devereux-Fitzgerald et al [48], however, it is argued that supporting self-regulation is also important for interventions targeting older adults, but specific characteristics of this population have to be taken into account. Older adults value maintaining social relations with others and rather focus on short-term health benefits instead of long-term benefits. The blended intervention reported here takes those aspects into account by extending community-based PA programs, where social relations already exist [30]. Furthermore, the intervention facilitates personal guidance of a coach. Finally, the goals revolve around activities of daily living that are recognizable for older adults, instead of general (long-term) health benefits. Examples are joining their spouse for gardening or being able to go for a walk with their grandchildren. Furthermore, one could argue that providing tools such as a tablet help older adults overcome their declining ability to self-regulate behavior. Daily and weekly schedules and reminders, for instance, lower the cognitive effort needed for action planning.

The reviews mentioned earlier [47,48] describe general PA interventions for older adults. Only a few review studies specifically focus on the role technology can play to promote PA in older adults [25,41,49]. eHealth interventions that explicitly take social aspects into account are rare. Notable early work of Silveira et al [50] describes a pioneering study in which a tablet not only supported self-regulation but also social support. Older adults could, for instance, monitor progress of other participants and could send each other motivational messages. Nevertheless, the study failed to demonstrate a beneficial effect of facilitating social support. The question remains, however, if merely facilitating online communication between peers who are not acquainted with each other is sufficient to capture the richness of social interactions. In contrast to the work of Silveira, the blended intervention described in this study is designed to extend existing community-based programs that rely on weekly face-to-face classes. Therefore, the proposed intervention can rely on social relations that are already in place.

As noted by Khaghani-Far et al [26], the present computer-generated support, mostly in the form of a virtual coach, is not capable of replacing the emotional support provided by a human coach. The contribution of our work is the demonstration of how PA in older adults can be stimulated by a blended approach. To our knowledge, only 1 prior study had combined the benefits of a face-to-face exercise program to the possibilities of mHealth. Lee et al [51] provided older adults with a tablet which they used for doing home-based exercises 3 times a week during a period of 8 weeks, in conjunction with weekly group-based exercises. Although participants showed an increase in motivation, no difference in physical functioning was observed. As suggested by the authors, a reason for this absence might be the limited number of participants in the trial (N=26) and the short duration of the study.

### Limitations

The ability for older adults to draw up a personal training schedule is a key element of the design. Users can choose from a library containing approximately 50 different functional exercises. Furthermore, the duration, number of repetitions, and the intensity level of each exercise can be manipulated, amounting to roughly 500 unique exercise variations. Despite the ability to personalize the exercise program in great detail, there are still limitations to the tailoring. Older adults with specific limitations will not be able to perform some exercises in the manner demonstrated in the app or they may prefer outdoor activities above the home-based functional exercises (ie, taking a walk, riding a bike, gardening, etc). The variation in individual preferences is virtually unlimited. To accommodate this, the app was designed with the ability to add user-defined activities to the personal training schedule. The app allows the user to plan, monitor, and evaluate those exercises but does not contain instructions or video demonstration. The support for user-defined exercises is therefore somewhat limited.

Another key element of the design is the ability to receive remote feedback from a coach. As pointed out, the support by a human is more rich and effective than computer-generated feedback. There is, however, also a drawback. To be effective, feedback needs to be timely. Automated feedback can be near instant. In contrast, depending on the availability of the coach, older adults will have to wait some time to receive personal feedback.

A final limitation is the need for validation. The work presented here is ongoing. In line with the MRC framework, the developed intervention has to be validated rigorously by feasibility and effectiveness studies. First, older adults were involved during the development of the intervention—initially by conducting focus groups to explore their attitudes toward a blended intervention and afterwards to pilot the exercises and tablet use. Due to practicalities, however, the exercises and tablet use were evaluated separately. Older adults that participated in the evaluation of the tablet focused on the usability of the app; they did not actually perform the exercises. In contrast, the older adults that evaluated the exercises did so without the support of a tablet. Thus, how users perform exercises at home supported with a tablet still needs to be addressed with a more extensive usability study.

Second, the effectiveness of the theoretically underpinned intervention has yet to be empirically determined. During the next phase of the study, an RCT will cast light on to which extent the intervention leads to increased adherence and health benefits in the long run. This will extend the findings of Lee et al [51] by testing the blended intervention among a sufficiently large number of older adults (N=240) for a 12-month period. In addition, the RCT will investigate how the effects of the exercise program can be reinforced by dietary intake [27].

### Conclusions

In summary, an evidence-based blended intervention was designed to promote PA amongst older adults. The underlying design choices are underpinned by behavior change techniques that are rooted in self-regulation. Key components of the tablet-supported intervention are a tailored program that accommodates individual needs, demonstrations of functional exercises, (self-)monitoring, and remote feedback. The blended approach combines the convenience of a home-based exercise program for older adults with the strengths of mHealth and personal guidance.

## Abbreviations

ADL: activities of daily living
BCT: behavior change technique
CALO-RE: Coventry, Aberdeen & London–Refined taxonomy
mHealth: mobile health
MRC: Medical Research Council
PA: physical activity
RCT: randomized controlled trial
